# Husband’s migration status and contraceptive behaviors of women: evidence from Middle-Ganga Plain of India

**DOI:** 10.1186/s12905-023-02325-z

**Published:** 2023-04-15

**Authors:** Ramkrishna Samanta, Jadab Munda

**Affiliations:** International Institute for Population Sciences, Govandi East, Deonar, Mumbai, Maharashtra 400088 India

**Keywords:** Contraception, Left behind wives, Family planning, Middle-Ganga plain

## Abstract

**Background:**

Male out-migration is negatively associated with contraceptive use in developing countries. This study aimed to examine the effect of male out-migration on the contraceptive behaviour of women in the Middle-Ganga Plain (MGP) region.

**Methods:**

The data has been collected from the Middle-Ganga Plain survey (2018–19), which was conducted by the International Institute for Population Sciences (IIPS). The overall sample size was 1314 wives left behind and 1402 non-migrant wives. Multivariate logistic regression analysis was used to examine the relationship between the variables. Statistical analyses were done using SPSS version 25.

**Result:**

The result shows that female sterilization was the most common method used by both left behind wives (30.9%) and non-migrant wives (34.6%). Most of the left-behind women didn't use contraception because their husbands were away from home (30.86%). The result also shows that left behind wives were less likely to use any methods of contraception than the non-migrant wives. Left behind women were more tended to use the modern methods (OR-0.71, 95%CI = 0.57–0.88) than any traditional methods (OR-0.61, 95% CI = 0.46–0.80). Age, religion, family type, working status, and marital duration were strongly associated with women's contraceptive use.

**Conclusion:**

These results strengthen the existing literature that explains how migration affects women's health. Therefore, there is an important need to develop and implement comprehensive education programs and policy on contraception use.

## Background

Maternal complications are the leading cause of mortality for women in developing nations, and modern birth control is one way to stop this [1]. According to World Health Organization (WHO), In 2017, over 810 women died every day from preventable causes associated to pregnancy and childbirth around the world, with an estimated 295,000 maternal deaths [2]. India has made significant efforts over the past few decades to foster an environment that is favourable for family planning (FP), where couples can deliberate choices regarding the timing of births, spacing between births, or limiting births through the use of contraceptives to achieve desired family size. This has been accomplished by making several significant policy and programmatic decisions [3, 4]. Despite decades of spending on family planning programs in India, a significant share of women, especially in regions like Bihar and Uttar Pradesh, don't use modern contraceptive techniques [5].

The reproductive health and contraceptive behavior of women is influenced by a variety of factors, including individual factors (education, economic status, race, ethnicity, place of residence, religion, occupation), demographic factors (age, sex, parity), relationship characteristics (partnership types, communication, attitude), family or household characteristics (family structure, co-residence, household economy, division of labor) etc. [6, 7]. Moreover, migration is a significant factor that have an impact on the contraception use among women. The previous studies shows that, male out-migration is negatively associated with reproductive health services and contraception in developing countries [5, 8]. A study conducted in Bihar suggests that male out-migration is one of the most significant factors that could have influenced the prevalence of modern contraceptives [9]. According to a study conducted in Nepal, women with migrant husbands were less likely to use contraception and have access to family planning services while having greater autonomy than those women staying together with their husbands [10]. In Nepal, the decline in total fertility was demonstrated to be significantly influenced by spousal separation in the absence of a comparable increase in the use of contraception [11]. Other studies conducted in Nepal demonstrate that contraceptive use depends on the frequency of the husband's visits to the family, as well as the duration and destination of migration [10, 12].

There are a lot of women who don't use modern contraceptive techniques, especially in places like Bihar and Uttar Pradesh. According to NFHS-5, about 43% of women in Uttar Pradesh and 44% of Bihar used modern contraceptive methods [13]. Empirical study in Bihar and other parts of India shows a number of reasons for contraceptive non-use, including side effects, difficulty in accessing family planning methods, and husband’s opposition [14, 15]. There has been limited research in Bihar and Uttar Pradesh specifically examining family planning issues in the context of migration, while there have been studies examining the effects of male out-migration on STD/HIV and utilization of maternal health facilities [16–18]. Therefore, this study examined the role of male out migration on the contraceptive use behavior of women in the Middle-Ganga Plain.

## Methods

### Study area

The middle-ganga plain has a massive geographical area (144,409 km^2^) and tremendous human, cultural, and economic significance, making it India's heart region. The Middle-Ganga Plain occupies the eastern section of Uttar Pradesh and Bihar and lies to the east of the Upper Ganga plain and details about it can be found in the Middle-Ganga Plain (MGP) report [19]. Middle-Ganga plain is known as one of India's most prominent regions of out-migration. Unemployment is the main reason for male out migration in Uttar Pradesh and Bihar. According to the Census of India (2011), Uttar Pradesh (12.32 million) and Bihar (7.45 million) have the highest out-migration rates. Census 2011 reported 10.11 million male migrants out from these two states [20]. It is important to record the reason for male out migration because there is insufficient documentation for evidence in public health [21].

### Sampling design

**Fig. 1:**
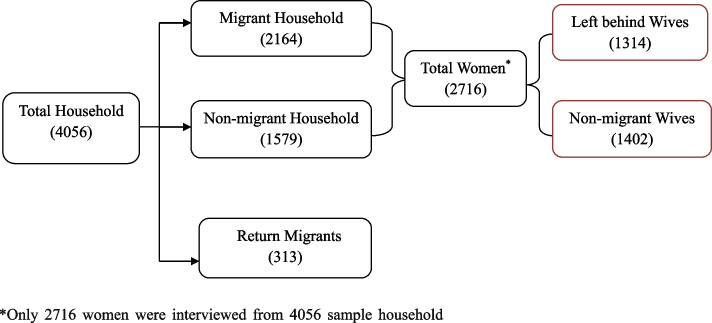
Framework for selecting the study sample, MGP survey, 2018–19 *Only 2716 women were interviewed from 4056 sample household

The data has been collected from the Middle-Ganga Plain survey (2018–19), which was conducted by the International Institute for Population Sciences (IIPS), Mumbai. It is a cross-sectional survey covering the entire Middle-Ganga Plain (MGP). A multi-stage stratified random sampling was used in this study. The sample population included Left Behind women and non-migrant wives aged 15 to 49 who lived in the Mid-Gangetic plain during the study period. This study defines left behind women as currently married women whose husbands have resided in a different country/state/district/block/town for at least one year for employment purposes. The total number of households in this study is 4056, comprising 1579 non-migrant households and 2164 migrant households. The total sample size for women is 2716, with 1314 wives left behind and 1402 non-migrant wives [19]. The details of the sample distribution are shown in Fig. 1.

### Outcome variable

The outcome variable of this study is ever used contraceptive methods, which is based on the question, "Which methods of family planning have you ever used?".

### Independent variable

This study also included a series of socio-demographic variable. These included respondents aged (15–24, 25–34, above 35), literacy (yes, no), caste (SC, ST, OBC, and others), religion (Hindu and Muslim), family type (nuclear and joint/extended), marital duration (less than 10 years, 10–20 year, and > 20 year), currently working (yes, no), Landholding ( landless, less than 1 acre, and more than 1 acre, Region (Uttar Pradesh and Bihar), Migration Duration (less than 5 years, 5–10 years, more than 10 years), frequency of husband’s home visit (no/once, 2–3 times, more than 3 times), Autonomy and decision making power (low, medium, and high), Remittance received (yes, no).

### Statistical analysis

Several statistical analyses are applied to assess the differences between Left behind and Non-Migrant Wives. First, chi-2 statistics is used to compare the left behind and non-migrant wives across various socio-demographic characteristics. Second, Bi-variate analysis is used to explain the dynamics of women's contraceptive behavior. Third, we employed multivariate logistic regression to examine the factors influencing women's contraceptive behavior. Statistical analyses were done using SPSS version 25.

## Result

### Socio-demographic characteristics

Table 1 represents the Socio-demographic characteristics of women. The results show that in the age group of 16–24 (18.7%) and 25–34 (47%), the population percentage of left-behind wives is higher than that of non-migrant wives; however, in the age group of > 35 (45.8%), the population percentage of non-migrant women is higher. The illiterate (54%) and 10–20 years of marital experience (38.5%), both the migrants and non-migrant wives’ populations are in higher concentration. In the case of the percentage of the work participation population, most women (78.3%) are engaged in non-working categories. The OBC (54.9%) social categories, Hindu religion (84.7%), Nuclear family types (60.6%), and the landless population represented higher percentages in both categories. In the region-wise division, Eastern Uttar Pradesh represented a higher percentage of non-migrant wives (54.4%), and Bihar represented a higher percentage of left-behind wives (56.2%).

**Table 1:** Socio-demographic characteristics of study population

Background Variable	Non-migrant Wives		left behind Wives		Total Women	
	**%**	**n**	**%**	**n**	**%**	**n**
Age group***						
16–24	13.3	185	18.7	244	15.9	429
25–34	40.9	574	47	616	43.9	1190
> 35	45.8	642	34.2	449	40.2	1091
**Social category**						
Scheduled caste	26.9	377	27.5	362	27.2	739
Scheduled tribe	3	42	2.1	27	2.5	69
OBC	54.7	766	55.1	720	54.9	1486
Others	15.4	216	15.3	200	15.4	416
Religion***						
Hindu	87.5	1226	81.7	1071	84.7	2297
Muslim	12.5	175	18.3	238	15.3	413
**Currently working**						
Yes	22.7	318	20.6	268	21.7	586
No	77.3	1082	79.4	1036	78.3	2118
**Literacy**						
Yes	45	628	47.2	610	46	1238
No	55	769	52.8	685	54	1454
Marital duration***						
< 10 year	24.8	346	30.9	403	27.7	749
10–20 year	38.5	540	43.4	568	40.9	1108
> 20 year	36.7	515	25.7	338	31.4	853
Family type**						
Nuclear	63.4	887	57.7	755	60.6	1642
Joint	36.6	513	42.3	553	39.4	1066
Land holding***						
Landless	49.2	689	55.2	721	52.1	1410
< =1 acre	28.6	401	30.1	394	29.3	795
> 1 acre	22.2	311	14.8	194	18.6	505
Region***						
Uttar Pradesh	54.4	763	43.8	575	49.3	1338
Bihar	45.6	638	56.2	734	50.7	1372
**Frequency of husband’s visit home**						
No/once			27	384		
2–3 times			62	656		
More than 3 times			11	106		
**Migration duration**						
< 5 years			29	414		
5–10 years			36	298		
>10 years			35	348		
**Total (n)**	**1402**		**1314**		**2716**	

Level of significance

^*^p < 0.05

^**^p < 0.01

^***^p < 0.001

### Knowledge of contraception

About 95% of women are aware of any contraceptive methods used (Table 2). Wives of migrants who were left behind (96%) were slightly more aware of using any contraception method than wives of non-migrants (94%). The result represents that both the non-migrant and left behind wives has more knowledge about female sterilization (92%) than the other modern contraceptive methods, whereas female condom (10%) is an uncommon modern contraceptive method among them. In the case of traditional methods, both the non-migrant and left behind wives have higher knowledge regarding Rhythm Methods (34%). In contrast, left-behind wives have higher knowledge about the Withdrawal method (29%) than non-migrant wives.

**Table 2:** Knowledge of contraceptive use among the left behind wives and non-migrant wives

Knowledge of Contraceptive Methods	Non-migrants Wives		Left behind Wives		Total Women	
	**%**	**n**	**%**	**n**	**%**	**n**
**Modern Methods**						
Female Sterilization	91	1014	93	988	92	2002
Male sterilization	70	749	66	703	68	1452
IUD	63	681	68	704	65	1385
Pill	72	780	73	768	72	1548
Injectable	59	641	62	639	61	1280
Emergency contraception	29	353	26	333	27	686
Condom/Nirodh	61	687	58	643	59	1330
Female Condom	11	100	9	94	10	194
Any modern methods	93.94	913	96.07	912	95.05	1825
**Traditional Methods**						
Rhythm Method	34	366	33	335	34	701
Withdrawal Method	27	347	29	341	28	688
Contraceptive herbs	10	94	9	101	9	195
Lactation Amenorrhea Method	6	59	4	41	5	100
Any traditional methods	45.88	127	44.36	106	44.85	235
Others	4	25	3	30	3	55
**Any contraceptive methods**	**94**	**1040**	**96**	**1018**	**95**	**2058**
**Total (n)**	**1093**		**1058**		**2151**	

### Use of contraception

About 38.6% of non-migrant wives and 32.6% left behind wives used any modern contraceptive method (Table 3). On the other hand, 13.84% of non-migrant wives and 12.01% of left-behind wives used the traditional method**.** Among the modern contraceptive methods, female sterilization was the most common method used by both left-behind wives (30.9%) and non-migrant wives (34.6%). Among the traditional contraceptive methods, the rhythm method was the most common method used by women.

**Table 3:** Ever used contraceptive methods among the left behind wives and non-migrant wives

Ever Used Contraceptive Methods	Non-migrants Wives		Left behind Wives		Total	
	**%**	**n**	**%**	**n**	**%**	**n**
**Modern Methods**						
Female Sterilization	34.6	327	30.9	254	32.7	581
Male sterilization	1.6	13	2.3	11	1.9	24
IUD	4.4	32	3.3	31	3.8	63
Pill	2	15	3.5	19	2.8	34
Injectable	2.3	12	1.4	11	1.8	23
Emergency contraception	2.1	12	3.2	6	2.7	18
Condom/Nirodh	15.1	137	11.3	96	13.1	233
Female Condom	7.5	6	5.3	7	6.5	13
Any modern methods use	38.62	389	32.65	312	35.44	701
**Traditional Methods**						
Rhythm Method	30.4	127	23.3	74	26.8	201
Withdrawal Method	22.8	101	21.4	80	22	181
Contraceptive herbs	3	6	5.9	7	4.4	13
Lactation Amenorrhea Method	1.6	3	0	1	0.9	4
Any traditional methods used	13.84	175	12.01	124	12.88	299
Others	12.8	5	3.1	2	8.5	7
**Any contraceptive method**	**52.46**	**564**	**34.62**	**436**	**48.36**	**1000**
**Total (n)**	**1093**		**1058**		**2151**	

### Regional pattern of contraception uses among the women

**Fig. 2:**
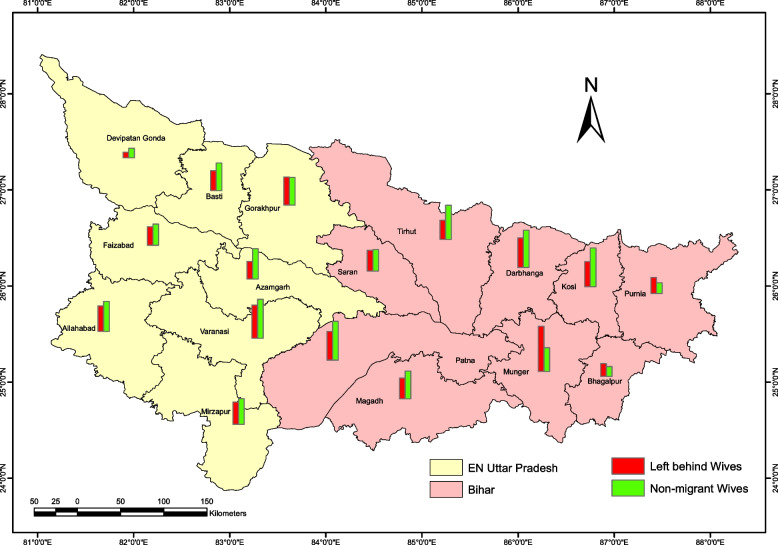
Division-wise modern contraceptive use among women in Middle-Ganga Plain

**Fig. 3:**
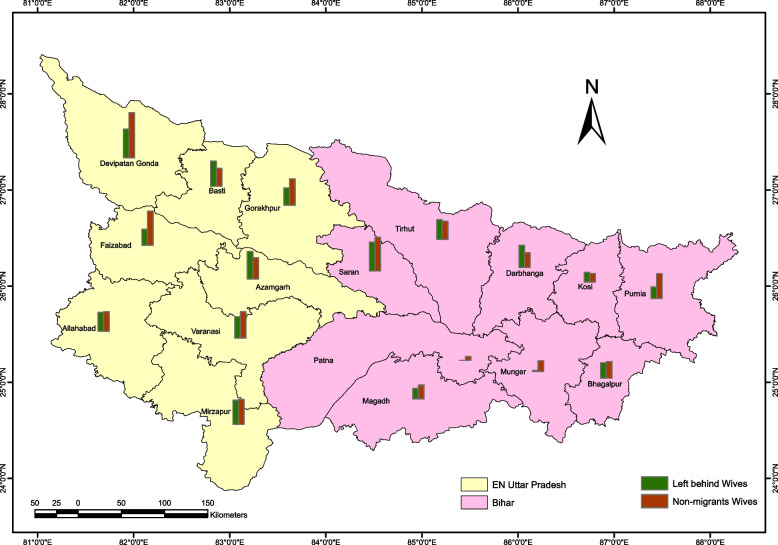
Division-wise traditional contraceptive use among women in Middle-Ganga Plain

The highest and lowest modern contraceptive use among left behind wives was found in Munger (63%) and Devipatan Gonda (8%) divisions, respectively (Fig. 2). Whereas the corresponding figure for non-migrant wives is 54% and 13% respectively found in Patna and Devipatan Gonda. Traditional methods were used highest in Saran for both left behind wives (21%) and non-migrant wives (24%) (Fig. 3). In contrast, Traditional methods used are lowest in Patna division for both left behind wives (0.1%) and non-migrant wives (3%).

### Reason for not using contraception

The most common reason for not using any contraceptive methods was the needs of children (Table 4). Approximately one-fourth of women reported that they want children. Among the left behind women, the most common reason for not using contraception was husband was away from home (30.86%). In contrast, the main reason for not using contraception among non-migrant wives was wants children (28.70%), followed by fear of side effects (10.85%). One-third of women stated that they had no reason for not using any form of contraception.

**Table 4:** Reason for not using any contraceptive methods

Reasons	Non-migrants Wives		Left behind Wives		Total Women	
	**%**	**n**	**%**	**n**	**%**	**n**
Husband away from home	0.36	3	30.86	212	17.21	215
Difficult to get/costly	6.88	23	1.43	8	3.87	41
Fear of side effect	10.85	60	3.52	23	6.8	83
Wants children	28.7	139	19.36	95	23.54	234
Against Religion	4.81	22	5.15	29	4.87	51
No reason	35.32	153	28.52	142	31.56	295
Others	13.37	73	11.15	42	12.14	115
**Total (n)**	**483**		**551**		**1034**	

### Prevalence of contraceptive use across the socio-demographic variables among the left behind wives and non-migrant wives

Table 5 represents the Prevalence of contraceptive use among women. The result shows that non-migrant wives are utilizing a higher percentage of modern and traditional contraceptive methods in the age groups of > 35 (52.27%) and 25–34 (52.04%), Whereas the left behind wives is using a higher percentage of both this contraceptive method in the age groups between 25–34. The Hindu religion groups women who use a higher percentage of both modern and traditional methods of contraception (39.26%). The working participation percentage of non-migrant women who are engaged in working categories (49.69%) and the left behind wives who are engaged in not-working categories (47.60%) used modern contraceptive methods. In the case of marital duration, non-migrant (53.34%) and left behind (45.36%) women who have > 10 years of marriage experience used modern contraceptive methods. The households who have < 1 acre of land are more likely to use modern contraceptive methods than other categories; whereas in the region classification, both Eastern Uttar Pradesh (34.5%) and Bihar (35.49%), non-migrant and left behind wives preferred to use of modern contraceptive methods. In the case of autonomy and decision-making factor, those women have high (44.68%) autonomy and decision-making power within the households they used a higher percentage of modern contraceptive methods, whereas non-migrant wives (47.3%) used modern methods highly than those left behind wives (43.39%).

**Table 5:** Prevalence of contraceptive use across the socio-demographic variables among the left behind wives and non-migrant wives

*Background Variable*	Non-migrant wives			Left behind wives			Total Women		
	**Modern**	**Traditional**	***P*-value**	**Modern**	**Traditional**	***P*-value**	**Modern**	**Traditional**	***P*-value**
**Age Group**									
16–24	6.22	13.95	0.000	10	7	0.000	15.19	7.21	0.000
25–34	41.52	52.04		50.56	61.52		35	16	
> 35	52.27	34.01		39.46	31.56		47	12	
**Social Category**									
Scheduled Caste	38.65	10.51	0.35	35.16	7.93	0.44	36.83	9.17	0.15
Scheduled Tribe	33.35	12.83		22.54	12.09		29.98	12.53	
OBC	41.77	15.56		33.88	11.44		37.87	13.4	
Others	27.85	13.31		27.34	20.27		27.57	17.09	
**Religion**									
Hindu	41.16	14.46	0.000	37.92	10.93	0.000	39.56	12.71	0.000
Muslim	20.34	9.34		13.3	15.97		15.71	13.71	
**Currently Working**									
Yes	49.69	13.78	0.000	28.41	12.1	0.000	31.94	12.91	0.000
No	35.64	14.05		47.6	11.71		48.56	12.79	
**Literate**									
Yes	36.72	13.97	0.84	29.16	13.83	0.14	32.73	13.8	0.23
No	39.98	14.05		36.42	10.42		38.16	12.18	
**Marital Duration**									
< 10 year	19.82	11.24	0.000	17.8	8.5	0.000	18.71	9.79	0.000
10–20 year	40.87	15.43		36.19	14.26		38.36	14.8	
> 20 year	53.34	14.07		45.36	12.47		49.56	13.31	
**Family Type**									
Nuclear	39.31	14.91	0.77	35.32	9.94	0.03	37.21	12.3	0.08
Joint	37.3	11.79		27.43	16.06		32.18	14	
**Land holding**									
Landless	33.63	12.09	0.000	28.91	8.74	0.000	31.07	10.27	0.000
< =1 acre	49.96	15.78		38.41	18.63		43.65	17.32	
> 1 acre	36.72	16.33		38.33	12.16		37.47	14.64	
**Region**									
Uttar Pradesh	39.01	18.83	0.003	30.88	15.96	0.003	34.5	17.59	0.000
Bihar	38.46	11.73		33.1	11		35.49	11.32	
**Autonomy and Decision making**									
Low	36.83	12.92	0.007	22.67	9.77	0.000	31.32	11.69	0.002
Medium	36.72	17.88		30.71	12.11			14.67	
High	47.3	8.15		43.39	13.76			11.91	
**Migration Duration**									
> 5 year				28.36	11.11	0.11			
**5–10 year**				27.4	11.39				
> 10 year				39.92	12.99				
**Frequency of husband’s home visit**									
**No/once**				31.66	14.92	0.10			
**2–3 times**				35.67	10.47				
> 3 times				31.68	9.23				
**Remittances Received**									
**No**				43.8	6.06	0.16			
**yes**				31.74	12.75				

### Determinants of contraception use among women

The multivariate logistic regression result (Table 6) shows that left-behind wives were less likely to use any methods of contraception than non-migrant wives. Left behind women tended to use modern methods more (OR-0.71, 95%CI = 0.57–0.88) than traditional methods (OR-0.61, 95% CI = 0.46–0.80). Women under the age group of 35 + have a higher probability of modern contraceptive use in total (OR-1.75, 95%CI = 1.17–2.61) and non-migrant (OR-3.06, 95%CI = 1.46–6.41) categories. The Scheduled Caste women are less preferred to use traditional contraceptive methods (OR-0.55, 95%CI = 0.35–0.87) than the other social groups. The religious categories show that Muslim women have less probability of contraceptive use in modern (OR-0.27, 95%CI = 0.19–0.39) and traditional (OR-0.64, 95%CI = 0.43–0.97) methods. The probability of modern contraceptive use was higher among the currently working women (OR-1.61, 95%CI = 1.25–2.09. The education of women plays an important role for enrich knowledge in the form of awareness about family planning. Where the result illustrated that literate non-migrant wife (OR-1.39, 95%CI = 1.01–1.92) are more likely to use modern contraceptive methods than illiterate non-migrant women The landholding of the women’s household is also a significant determinant factor for the use of contraceptive methods. Women with less than 1 acre of land were more likely to use any modern contraceptive use (OR-1.60, 95%CI = 1.25–2.05), whereas the left behind wives (OR-1.88,95%CI = 1.22–2.90) having a higher probability of use than non-migrant wives (OR- 1.51, 95%CI = 1.06–2.14). The left-behind wives who stay with joint families are less likely to use modern contraception methods (OR-0.65, 95%CI = 0.44–0.98). In the case of marital experience, the women who have more than 20 years of marital experience are significantly using both modern (OR-2.28, 95%CI = 1.43–3.62) and traditional (OR-2.00, 95%CI = 1.10–3.63) contraceptive methods; whereas left behind women having a higher probability of using modern contraceptive methods (OR-2.86, 95%CI = 1.28–6.36) than non-migrant women. The women who have medium autonomy and decision-making right within the household they are more prefer to use traditional contraceptive methods (OR- 1.58, 95%CI = 1.14–2.20); whereas the left-behind wives (OR- 2.39, 95%CI = 1.23–4.65); have high autonomy and decision-making right within the household, they also prefer to use modern methods than non-migrant wives. The Bihar represent that women are using significantly less traditional contraceptive methods (OR-0.54, 95% CI = 0.40–0.72) than in Eastern Uttar Pradesh, and the left behind wives (OR-0.50, 95%CI = 0.29–0.86) in Bihar is less like used traditional contraceptives methods than non-migrant women (OR-0.54, 95%CI = 0.36–0.80).

**Table 6:** Determinants of contraceptive use among the left behind wives and non-migrant wives

Background Variable	Non-migrant wives		left behind wives		Total Women	
	**Modern**	**Traditional**	**Modern**	**Traditional**	**Modern**	**Traditional**
	**OR [CI]**	**OR [CI]**	**OR [CI]**	**OR [CI]**	**OR [CI]**	**OR [CI]**
**Migration Status**						
Non-migrant wives®						
left behind wives	1	1	1	1	0.71**[0.57–0.88]	0.61***[0.46–0.80]
**Age Group**						
16–24®						
25–34	2.29**[1.28–4.09]	1.08[0.56–2.10]	1.68[0.86–3.29]	2.43[0.98–6.05]	1.75**[1.17–2.61]	1.37[0.85–2.23]
> 35	3.06**[1.46–6.41]	0.75[0.30–1.87]	1.54[0.64–3.70]	1.61[0.50–5.17]	1.86*[1.11–3.11]	0.98[0.51–1.88]
**Social Category**						
Others®						
Scheduled Tribe	0.72[0.28–1.82]	0.47[0.14–1.59]	1.42[0.37–5.83]	0.84[0.15–4.71]	0.7[0.33–1.46]	0.67[0.27–1.62]
Scheduled caste	1.06[0.62–1.79]	0.65[0.35–1.22]	0.75[0.38–1.48]	0.55[0.24–1.23]	0.77[0.53–1.13]	0.55*[0.35–0.87]
OBC	1.35[0.86–2.11]	0.9[0.54–1.52]	0.88[0.48–1.62]	0.80[0.40–1.56]	0.99[0.71–1.37]	0.77[0.52–1.12]
**Religion**						
Hindu ®						
Muslim	0.35***[0.21–0.58]	0.53*[0.29–0.97]	0.15***[0.07–0.29]	0.97[0.52–1.81]	0.27***[0.19–0.39]	0.64*[0.43–0.97]
**Currently working**						
No®						
Yes	1.52*[1.06–2.19]	1.35[0.84–2.17]	1.99**[1.25–3.15]	1.62[0.86–3.05]	1.61***[1.25–2.09]	1.41[0.99–1.99]
**Literacy**						
No®						
Yes	1.39*[1.01–1.92]	1.12[0.75–1.67]	1.03[0.70–1.52]	1.17[0.71–1.89]	1.14[0.91–1.42]	1.08[0.81–1.45]
**Marital Duration**						
< 10 year®						
10–20 year	1.70*[1.07–2.72]	1.7[0.96–3.02]	2.30**[1.30–4.06]	1.84[0.92–3.69]	2.00***[1.44–2.79]	1.60*[1.06–2.40]
> 20 year	1.75[0.91–3.34]	2.24[0.97–5.13]	2.86*[1.28–6.36]	2.05[0.73–5.73]	2.28***[1.43–3.62]	2.00*[1.10–3.63]
**Family Type**						
Nuclear®						
Joint	1.01[0.73–1.39]	0.93[0.62–1.39]	0.65*[0.44–0.98]	1.22[0.69–2.13]	0.91[0.72–1.16]	1.13[0.84–1.54]
**Land holding**						
Landless®						
<= 1 acre	1.51*[1.06–2.14]	1.25[0.79–1.96]	1.88**[1.22–2.90]	1.34[0.76–2.37]	1.60***[1.25–2.05]	1.3[0.94–1.82]
> 1 acre	1.06[0.69–1.60]	1.12[0.67–1.86]	1.62[0.90–2.89]	1.43[0.70–2.90]	1.18[0.86–1.62]	1.21[0.82–1.78]
**Autonomy and Decision making**						
Low®						
Medium	0.92[0.66–1.30]	1.65*[1.10–2.47]	1.15[0.68–1.96]	1.88[0.93–3.80]	1.04[0.81–1.35]	1.58**[1.14–2.20]
High	1.11[0.75–1.65]	0.81[0.46–1.42]	1.51[0.90–2.51]	2.39*[1.23–4.65]	1.3[0.98–1.71]	1.37[0.94–1.99]
**Region**						
Uttar Pradesh®						
Bihar	1.06[0.78–1.45]	0.54**[0.36–0.80]	1.14[0.76–1.71]	0.50*[0.29–0.86]	1.07[0.86–1.34]	0.54***[0.40–0.72]
**Migration Duration**						
> 5 year®						
5–10 year			0.87[0.55–1.39]	1.00[0.55–1.82]		
> 10 year			1.03[0.66–1.63]	0.94[0.50–1.78]		
**Frequency of husband’s home visit**						
No/once®						
2–3 times			1.34[0.90–1.99]	0.95[0.58–1.55]		
> 3 times			0.71[0.29–1.70]	0.61[0.19–1.93]		
**Remittance Received**						
No®						
yes			0.70[0.28–1.75]	1.99[0.40–9.76]		

Level of significance

^*^p < 0.05

^**^p < 0.01

^***^p < 0.001

® reference Category

## Discussion

This study examined the utilization of contraceptive methods among the non-migrant and left behind wives in the Middle Ganga Plain of Eastern Uttar Pradesh and Bihar; however, the study covers the topic related to the Knowledge, Ever-Used, Reason for Not Using and use of any modern and traditional contraceptive methods among the non-migrant and left behind wives. According to our study findings, most women know and prefer to use female sterilization of modern contraceptive methods, which is consistent with previous studies and it might be the reason for the husband’s knowledge, positive attitudes, autonomy and decision making power and perception regarding the family planning process in recent times [22–24]. However, among the individual groups, left behind wives have more knowledge about modern contraceptive methods such as IUDs, Pills, and Injectables, whereas they preferred to use Male Sterilization, Pills, and Emergency contraception methods than non-migrant women. This is due to the transfer of significant social remittances from the destination place, which acts as a robust transitional network to operate in the origin places [25]. The regional pattern of modern contraceptive use among left behind women and non-migrant women is higher in districts of Munger and Patna. This is due the substantial economic growth, increase of female literacy rate that push the left behind women to promote more decision making power for utilization of contraceptives methods in this regions [26–28]. Whereas the left behind women and non-migrant women are less used modern contraceptive methods in district of Devipatan Gonda. This might be reason of low literacy rate of the district specially female, having low economic growth rate, and they also come under backward regions grant fund (BRGF) of cabinet committee on economic affairs [29, 30]. In case of traditional methods of contraception Saran district having higher use and the Patna having lower use. This is due to the socio-economic impact of society and regional level development. At the individual level, the husband’s away from home, difficulty getting contraceptives, fear of side effects, wanting children, and against religion were identified as barriers to contraceptives. This study reveals that the most common reason for not using contraception among the left behind wives was the husband's away from home. Due to the husband’s absence at home, wives did not feel the need to use contraceptives because their husbands resided elsewhere. Their husband’s migration act as a natural method of contraception. Undoubtedly, because of the social circumstances that exist in rural communities, women may feel internally stigmatized to access family planning procedures in the absence of the husband [5, 9]. On the other side, the main reason for not using contraceptives among the non-migrant wives was the desire for children. Economic factors drive their preference for children. Many people want children because there would be more hands available to support the family with income.

There have diverse determinant factors for the use of contraceptive methods among the women in the Middle Ganga plain of Eastern Uttar Pradesh and Bihar. But male out migration is an important factor in Middle-Ganga plain that influenced the contraceptive behavior of women. This study found that left behind wives are less likely to use the any of the contraceptive methods than the non-migrants wives; this might be the reason for the preference for a male child, absence of interspousal communication, and poor outreaches of local ASHA workers [5, 9]. A study conducted in Pakistan shows that, due to a lack of communication between spouses over family planning, most were unaware of their partner's preference for family size and contraceptive use [31]. In the categories of the 35 + age group, non-migrant wives are more likely to use any modern contraceptive methods because after age groups of 35 + , they do not want to take any more children or the age groups of the parents become too older for their child parents age group ratio for future perspectives. Whereas the in the social categories groups, the Scheduled caste women are not so much interested in any contraceptive methods because most of the migrating husbands are from these social categories in India [32, 33]; due to this reason, they are unable to communicate with their wives regularly [5]. Our result also illustrated an exciting finding that Muslim women are less used to preferring any contraceptive method; this is due to the Muslim women thought that their religious dis approvals regarding the use of contraception and also get inspiration from the religious leader for not using any contraceptive methods [24, 34]. This study also found that Muslim women are more tend to use the traditional contraceptive methods than the modern methods. Because many Muslim women considered the traditional (also known as the 'Islamic method') method as a secure choice for spacing pregnancies because it is recommended by their religious doctrine and thus could not have negative effects on individuals [31]. Women who currently working in any sector highly used to prefer modern contraceptives; this might be the cause those who work in the different economic sectors get exposed to information related to contraceptive methods from the colleague and come to know about governmental policies like target free approach in 1996 and National Population policy in 2000 [22]. The landholding of a women’s household plays a significant role in using any contraceptive methods. The result shows that the left behind wives with less than 1 acre of land prefer any modern contraceptive methods than non-migrant wives; this might be the reason for women's empowerment and decision-making power among the left behind women. On the other hand our study result illustrates similar findings to previous studies that those women have high autonomy and decision making power within the household or society they are more preferred to use any contraceptive methods [24, 35, 36]. Family type is an important determinant factor for the use of contraceptive methods in India. In contrast, our finding shows that the left behind wives who live with joint families are less likely to use any modern contraceptive methods because of family pressure of wanting children and less communication of migrated husbands for sexual relationships. In the case of marital duration, the left-behind women who have more than 20 years of marriage experience they are more preferred to use any modern contraceptive methods than the non-migrant women; this is because after those years of marriage, they get fulfil their children's sex composition or they have at least one son, and one daughter [37]. The Bihar shows significant findings that the left behind wives is less like to use any traditional contraceptive methods than the non-migrant women in the Middle Ganga Plain region; this might be a positive reason for husband migration and husband get more exposed of television, radio, and interpersonal communication from the destination places [22].

### Study limitation

The study has some limitations: Firstly, only married women were interviewed for the study. Other possible respondents, such as health workers, ASHA workers, etc., are not interviewed. Secondly, the study did not perform any in-depth interviews, which helped in a better understanding. Thirdly, the data does not include a number of important predictor variables, including mass media exposure, the sex composition of the child, and others which have been found to have a direct impact on women's contraceptive behavior.

### Recommendation for further study

We don't know much about the contraception needs of migrants and their partners. The MGP surveys don’t give a good picture of the desire for children, unmet need for family planning, and current use of contraception. Therefore, in-depth qualitative research is required to better understand fertility desires, facilitators, and challenges to contraceptive usage among wives of labor migrants in the Middle-Ganga Plain.

## Conclusion

This study explain how migration affects women's reproductive health. The significant results of this study support the previous studies that male out or husband’s migration negatively affects contraception use among the left behind women. Several awareness programs and policies have been implemented for contraceptive use and reproductive health of women. But it is important to regularly monitor policy and programs at the individual level. To enhance the monitoring process, the government should strengthen the health workers such as ASHA (Accredited Social Health Activist), ANM (Auxiliary Nurse Midwife), and AWW (Anganwadi Workers). The community healthcare workers have a significant role in promoting reproductive health services as well as prenatal and postnatal care for women and children.

## Data Availability

This study was conducted by the International Institute for Population Sciences (IIPS) in Middle-Ganga plain using a large dataset publicly available on the MGP website (https://iipsindia.ac.in/content/mgp-report) with ethical standards being followed, including informed consent being obtained by all participants.

## Abbreviations

MGP: Middle-Ganga plain
OR: Odds ratio
CI: Confidence interval
